# Expression of DNA methyltransferases in adult dorsal root ganglia is cell-type specific and up regulated in a rodent model of neuropathic pain

**DOI:** 10.3389/fncel.2014.00217

**Published:** 2014-08-08

**Authors:** Sarah L. Pollema-Mays, Maria V. Centeno, A. V. Apkarian, Marco Martina

**Affiliations:** Department of Physiology, Northwestern University Feinberg School of MedicineChicago, IL, USA

**Keywords:** neuropathic pain, DNA methylation, DNMT expression, dorsal root ganglia, gene expression, SNI model

## Abstract

Neuropathic pain is associated with hyperexcitability and intrinsic firing of dorsal root ganglia (DRG) neurons. These phenotypical changes can be long lasting, potentially spanning the entire life of animal models, and depend on altered expression of numerous proteins, including many ion channels. Yet, how DRGs maintain long-term changes in protein expression in neuropathic conditions remains unclear. DNA methylation is a well-known mechanism of epigenetic control of gene expression and is achieved by the action of three enzymes: DNA methyltransferase (DNMT) 1, 3a, and 3b, which have been studied primarily during development. We first performed immunohistochemical analysis to assess whether these enzymes are expressed in adult rat DRGs (L4–5) and found that DNMT1 is expressed in both glia and neurons, DNMT3a is preferentially expressed in glia and DNMT3b is preferentially expressed in neurons. A rat model of neuropathic pain was then used to determine whether nerve injury may induce epigenetic changes in DRGs at multiple time points after pain onset. Real-time RT PCR analysis revealed robust and time-dependent changes in DNMT transcript expression in ipsilateral DRGs from spared nerve injury (SNI) but not sham rats. Interestingly, DNMT3b transcript showed a robust upregulation that appeared already 1 week after surgery and persisted at 4 weeks (our endpoint); in contrast, DNMT1 and DNMT3a transcripts showed only moderate upregulation that was transient and did not appear until the second week. We suggest that DNMT regulation in adult DRGs may be a contributor to the pain phenotype and merits further study.

## INTRODUCTION

Neuronal cell bodies located in the dorsal root ganglia (DRGs) generate the fibers that convey information from the skin, muscles and joints to the spinal cord (Gardner et al., 2000). This information includes signals mediated by activation of nociceptors (Ringkamp and Meyer, 2009). Nociceptive fibers can be myelinated A fibers, originating from large DRG neurons, or unmyelinated C fibers originating from small and medium DRG neurons.

In normal conditions nociceptors have a high firing threshold, and these neurons do not fire action potentials in the absence of noxious inputs. Only when the innervated organs are exposed to noxious stimuli are action potentials generated in the peripheral axonal endings. In the presence of nerve injury or chronic pain, however, nociceptors become intrinsically firing and their intrinsic firing frequency correlates with spontaneous pain (Weng et al., 2012); moreover, DRG neurons become capable of ectopic (somatic) firing and this is also considered a cause of neuropathic pain (Nordin et al., 1984; Sukhotinsky et al., 2004). These changes are often indicated as peripheral sensitization and they are not restricted to the injured nerve fibers but often include adjacent neurons, which may also generate spontaneous firing (Michaelis et al., 1996). These aberrant electrophysiological phenotypes are the consequence of large changes in ion channel gene expression and distribution (Novakovic et al., 1998; Tanaka et al., 1998; Dib-Hajj et al., 1999; Tan et al., 2006; Xiang et al., 2008; Fan et al., 2011; Weng et al., 2012).

Changes in ion channel expression are well documented and may explain the increased electrical excitability of DRG neurons, yet, one of the key issues that remain unaddressed concerns the mechanisms responsible for maintaining this altered gene expression that contributes to the chronification of pain. DNA methylation represents one of the most widely used mechanisms of enduring cellular memory and recently it was shown to mediate transient changes in gene expression in the hippocampus as well as long term changes in the cortex that occur during memory processes such as contextual fear conditioning (Miller and Sweatt, 2007; Miller et al., 2010). DNA methylation occurs on CpG sites by the action of enzymes known as DNA methyltransferases (DNMTs). Methylation is implicated in X chromosome inactivation (Wolf et al., 1984; Gendrel et al., 2012; Dupont and Gribnau, 2013) and is associated with gene silencing in general (Wolffe and Matzke, 1999), most likely by attracting methylation-specific transcriptional inhibitors. In DNA from mammalian somatic tissues ~70% of all CpG sites are methylated (Ehrlich, 2003). It is widely believed that active demethylation also takes place (Bruniquel and Schwartz, 2003; Barreto et al., 2007; Ooi and Bestor, 2008; Kaas et al., 2013), although no demethylase has been isolated so far. 5 DNMTs have been identified to date, although only DNMT1, DNMT3a, and DNMT3b possess catalytic activity (Bestor et al., 1988; Okano et al., 1998; Chedin, 2011; Qin et al., 2011). Interestingly, while the developmental importance of methylation was apparent from the start, its importance in the adult nervous system has been demonstrated only recently; knockout of DNMT1 and DNMT3a in adult forebrain neurons leads to several deficits in neuronal plasticity (Feng et al., 2010). An increasingly large number of papers are addressing the role of DNMTs in cancer and, more recently, in early life experiences (McGowan et al., 2009; Szyf and Bick, 2013), memory processes (Miller and Sweatt, 2007; Miller et al., 2010), addiction (LaPlant et al., 2010), and Schizophrenia (Grayson et al., 2005; Veldic et al., 2005). However, little work has been done thus far investigating the potential role of these enzymes in pain.

Hints that epigenetic changes in the spinal cord may be involved in pain chronification have come from reports that expression of DNMT’s is modulated in dorsal horn neurons following inflammation or nerve injury (Tochiki et al., 2012) and intrathecal spinal injection of a DNMT inhibitor improved pain behavior in the chronic constriction injury (CCI) model (Wang et al., 2011). Two recent reports of changes in histone acetylation in DRGs of a mouse model of neuropathic pain suggest that epigenetic mechanisms may also be important in maintaining DRG phenotype (Uchida et al., 2010a,b). However, no data are available concerning DNMT expression in adult DRGs or its potential modulation by pain. Here we show that DNMTs are expressed in adult DRGs and that expression of isoform DNMT3b is upregulated almost fourfold after nerve injury, while DNMT1 and DNMT3a are upregulated moderately and only transiently. This suggests a potential role for changes in DNA methylation in initiation and/or maintenance of the alterations in gene expression in DRGs in animal models of pain.

## MATERIALS AND METHODS

### ETHICS STATEMENT

All studies were approved by the Animal Care and Use Committee of Northwestern University.

### SPARED NERVE INJURY MODEL

Twenty to 21-day-old rats were anesthetized using gas anesthesia (isoflurane 1–2%, 30% N_2_O, and 70% O_2_). For induction of spared nerve injury (SNI)-neuropathy, the sciatic nerve of the left paw was exposed at the level of the trifurcation into the sural, tibial, and common peroneal nerves. The tibial and common peroneal nerves were tightly ligated and severed, leaving the sural nerve intact (Decosterd and Woolf, 2000). A second group of animals was used for control sham surgery. In this case, the left sciatic nerve was exposed just as in the SNI procedure but was not further manipulated.

### BEHAVIORAL TEST FOR TACTILE SENSITIVITY

Tactile sensitivity of the spared region of the operated paws was measured from the withdrawal responses to mechanical stimulation with von Frey filaments. Animals were placed in a cage with wire grid floor and allowed to habituate to the environment for 15 min. Filaments (Stoelting) of varying forces were applied to the plantar surface of the hind paw in ascending order. Each filament was applied for a maximum of 10 s. Paw withdrawal during the application was considered a positive response. 50% response thresholds were calculated according to (Chaplan et al., 1994). All animals that underwent SNI surgery developed tactile allodynia in the left paw that persisted until they were sacrificed.

### RT-PCR ANALYSIS

L4 and L5 DRGs were dissected from sham operated and SNI rats; DRGs both ipsilateral and contralateral to the injury were collected 1, 2, and 4 weeks after onset of SNI injury and frozen in liquid nitrogen and stored at -80°C. RNA was extracted using a Qiagen RNeasy RNA extraction kit that includes a column that binds DNA while allowing RNA to flow through in order to prevent DNA contamination. Sufficient RNA quantity and purity for each sample was verified by RNA Nanodrop measurements. RNA was then reverse transcribed into cDNA using Roche’s First Strand cDNA Synthesis kit and oligo dT primers. Quantitative RT-PCR was performed using a Roche Lightcycler 480 (LC480) with Roche probes or SYBR green master mix, primers (0.4 μM), and DRG cDNA. All PCR reactions were run using a 5 min hot start at 95°C, followed by 45 cycles of 10 s at 95°C, 15 s at 60°C, and 10 s at 72°C. All samples were run in duplicate. Gel electrophoresis (1.8% agarose) demonstrated a single band for amplification targets. Although all primers were intron spanning, cDNA negative and reverse transcriptase negative controls were done for genes of interest and were negative. GAPDH was used as the reference gene and all genes of interest were normalized to it. As recommended in the MIQE qRT-PCR guidelines, β actin transcript was quantified relative to GAPDH (data not shown) to validate the stability of GAPDH as a reference gene (Bustin et al., 2010). Standard curves were also done to calculate reaction efficiency for each gene product using dilutions of cDNA. All data were efficiency corrected using Roche LC480 software and the delta delta Ct method (Schmittgen and Livak, 2008). For the gene expression analysis 1, 2, and 4 weeks post surgery *n* = 8, 4, and 6 SNI/sham pairs, respectively. All primers and efficiencies are listed in **Table 1**.

**Table 1:** Primer sequences, efficiencies, slopes, and Roche probes used for genes of interest.

Gene	Primers		Efficiency	Slope	Roche probe and product #
DNMT1	L ccatcacgtctcacttcaagg	R tgcgtttcttatcctggtctc	1.99	-3.36	NA-CYBR green
DNMT3a	L ctgatgacgagcccgagtat	R ctgtcatccaccaagacacaa	1.96	-3.44	NA-CYBR green
DNMT3b	L gatgatcgacgccatcaag	R cgagcttatcattctttgaagcta	1.98	-3.37	8404689089001
GAPDH	L ctgcaccaccaactgcttag	R tgatggcatggactgtgg	2.00	-3.32	Rat GAPDH Ref 05046220001
Beta actin	L aaggccaaccgtgaaaagat	R accagaggcatacagggaca	1.91	-3.56	NA-CYBR Green

For the experiment determining relative abundance of DNMT’s in DRGs 5 naïve rats aged 4 weeks were used. L4 and L5 DRGs were dissected, frozen in liquid nitrogen and transcript levels quantified via qRT-PCR with CYBR green master mix as described above. As there was no significant difference in DNMT expression on the left or right side of the rats, data from the right and left DRGs from each rat were pooled and relative abundance determined.

### IMMUNOHISTOCHEMISTRY

Five to seven week-old naïve or SNI male Sprague Dawley rats were anesthetized using ketamine/xylazine (80 and 10 mg/0.1 kg, respectively) and perfused transcardially with saline and then with 4% paraformaldehyde. L4 and L5 dorsal DRGs were dissected out and post fixed in paraformaldehyde for 1 h after which they were cryoprotected with 10% sucrose overnight followed by 20% sucrose. DRGs were then embedded in OCT, frozen on dry ice, and cut into 20 μm slices using a Microtome Cryostat HM 505E. Sections were incubated at 40°C for 15 min prior to staining and then incubated for 36 h in primary antibody in TBS with 0.2% triton-X and 3% NGS. Sections were then washed with TBS and incubated for 2 h at 22–24°C in secondary antibodies in TBS. Sections were again washed (6–8 times) in TBS with one final wash in PBS. Sections were mounted and imaged on a Zeiss UV LSM 510 META confocal microscope. Z-stacks (8–12 in 2 micron steps) were taken for all image analyses. Primary antibodies were DNMT1 (Santa Cruz #20701; Sharif et al., 2007; Metivier et al., 2008; DNMT3a, Santa Cruz #20703; Ooi et al., 2010; Zhang et al., 2011a; Zhu et al., 2012; DNMT3b, Santa Cruz #20704; Di Giaimo et al., 2005; Zhang et al., 2011a). Secondary antibody was Invitrogen Goat anti-Rabbit 488. Controls including primary antibody alone and secondary antibody alone were negative. Additionally, specificity for DNMT1 and DNMT3a antibodies was verified using western blot (WB) analysis. The DNMT3b antibody did not give a detectable signal in WB, most likely because of the very low expression level of this molecule in adult DRG tissue.

### STATISTICAL ANALYSIS

Statistical significance of differences in expression data was determined at the 0.05 level using Analysis of Variance with *post hoc* correction (Student-Newman-Keuls). Data from sham left and right DRG were pooled and used for comparison with contra- and ipsi-lateral SNI. The “sham” expression level in the figures represents these pooled data. All data in the paper are presented as average ± standard error of the mean (SEM). Error bars in figures also represent SEM.

## RESULTS

### DNMT1 EXPRESSION IS ROBUST AND WIDESPREAD IN ADULT DRGs

**FIGURE 1:**
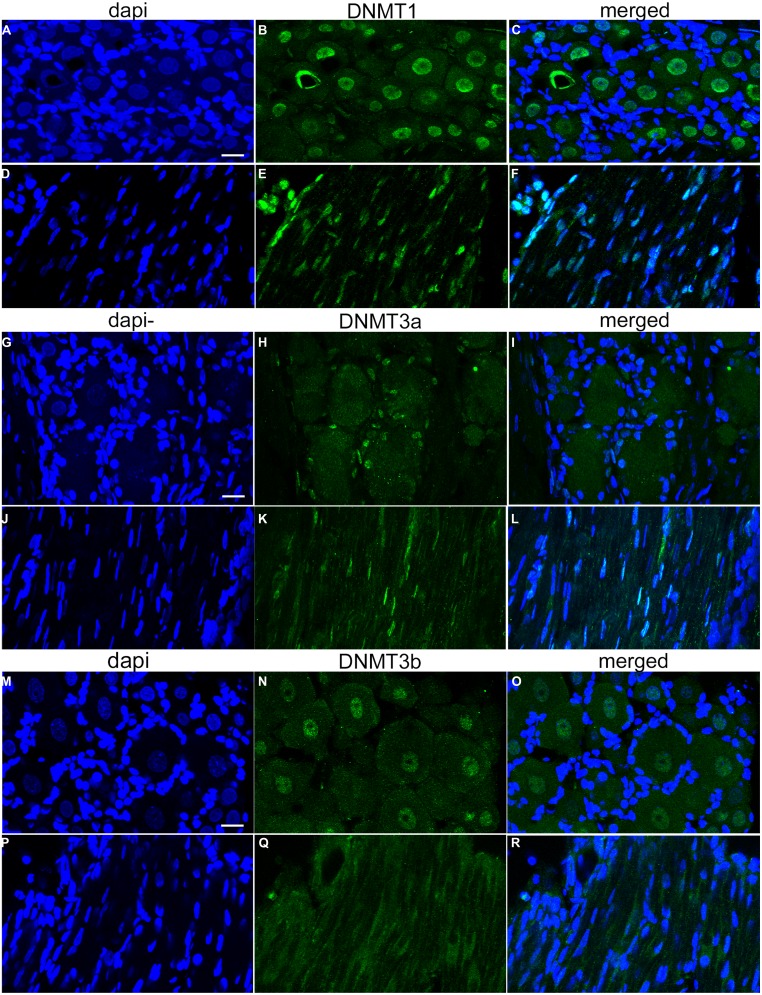
**DNA methyltransferase expression in DRG is cell-type specific.** 20-micron L4 and L5 DRG sections from control rats aged 5–7 weeks were immuno-labeled with DNMT antibodies as well as incubated with the nuclear labeling dye 4’,6-diamidino-2-phenylindole (DAPI). **(A–C)** Show robust and ubiquitous labeling of DNMT1 in nuclei of DRG neurons. Notably, there is little expression of DNMT1 detected in the satellite cells surrounding DRG neurons. In addition, widespread overlap of DAPI and DNMT1 was detected in axons, demonstrating ubiquitous expression in Schwann cells **(D–F)**. **(G–I)** In contrast to DNMT1, DNMT3a was not detected in neuronal nuclei but rather in satellite cells surrounding DRG neurons. **(J–L)** DNMT3a also showed widespread overlap with DAPI in nuclei of Schwann cells. **(M–O)** DNMT3b was detected in nuclei of all DRG neurons. **(P–R)** Notably, DNMT3b staining did not reveal overlap with DAPI in DRG axons, suggesting DNMT3b is not expressed in glia. Scale bar: 20 μM.

Immunochemical analysis was performed on 20 μm-thick slices obtained from L4 and L5 DRGs of 5–7 week old naïve rats to study the expression of the DNMT1, DNMT3a, and DNMT3b in L4 and L5 DRGs of adult rats. Immunostaining of DNMT1 demonstrated that expression is robust and is detected ubiquitously in the nuclei of DRG neurons as well as in Schwann cells (**Figure 1**). Indeed labeling with the nuclear dye 4′,6-diamidino-2-phenylindole (DAPI) revealed almost perfect overlap with DNMT1 expression in both DRG neuronal nuclei (**Figures 1A–C**, see arrowheads for examples) and the nuclei of Schwann cells surrounding DRG axons (**Figures 1D–F**). Interestingly, DNMT1 expression was not detected in the satellite cells that surround and support DRG neuronal cell bodies (**Figures 1A–C**).

### DNMT3a EXPRESSION IS FOUND IN GLIA OF ADULT DRGs

In contrast to DNMT1 staining in nuclei of DRG neurons, immunostaining L4 and L5 DRG sections for DNMT3a revealed robust expression in the nuclei of supporting satellite glia (**Figures 1G–I**). In addition DNMT3a was detected in the nuclei of many Schwann cells (**Figures 1J–L**). Thus, surprisingly, DNMT3a was not detected in nuclei of DRG neurons (**Figures 1G–I**).

### DNMT3b EXPRESSION IS LIMITED TO DRG NEURONS

Labeling of L4 and L5 DRG sections for DNMT3b demonstrated expression of DNMT3b in nuclei of all DRG neurons (**Figures 1M–O**). Notably, no overlap was detected for DNMT3b and DAPI in axons, suggesting that DNMT3b expression is selective for neurons and is absent in glia (**Figures 1P–R**).

### RELATIVE ABUNDANCE OF DNA METHYLTRANSFERASE TRANSCRIPT IN DRGs IN NAIVE RATS

**FIGURE 2:**
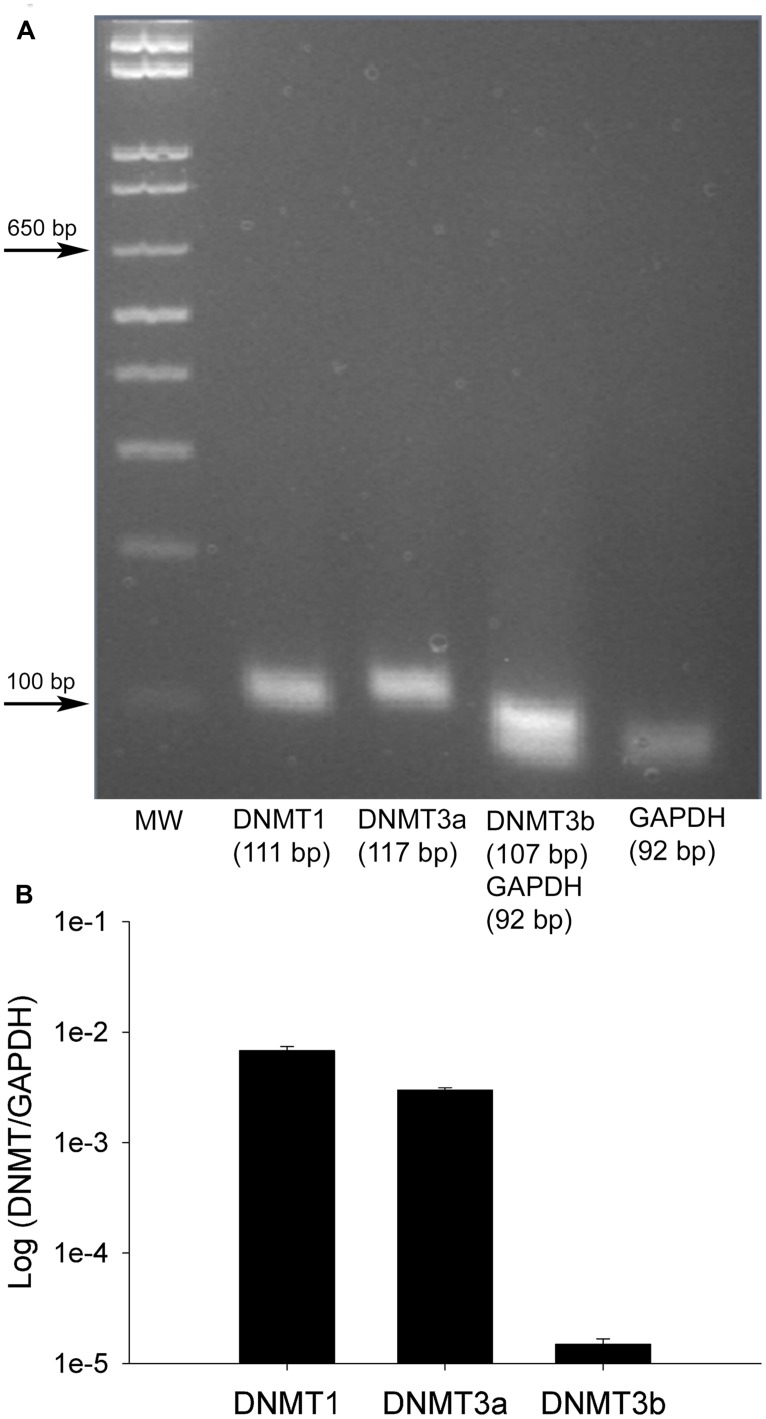
**Relative abundance of DNMT transcripts in adult rat DRGs. (A)** PCR products run on a 1.8% agarose gel demonstrate a single amplification product for each gene of interest. DNMT3b and GAPDH were run together as this assay involved multiplexing DNMT3b and GAPDH in the same PCR well, thus the two bands in one lane. **(B)** Quantitative RT-PCR was first used to determine the relative abundance of DNMTs from five naive rats at 1 month of age. All DNMT gene expression is normalized to GAPDH. Note the higher expression levels of DNMT1 and DNMT3a while DNMT3b is lower, consistent with immunocytochemistry data (**Figure 1**). Note the plot is on a logarithmic scale.

As no data are available concerning DNMT expression in rat DRG, we next quantified the relative abundance of DNMTs in L4 and L5 DRGs from naive rats. One month old rats were used to perform qRT-PCR measurements of DNMT transcript. qRT-PCR data was efficiency corrected, normalized to the GAPDH reference gene, and expression levels compared. PCR products were first run on a gel to demonstrate primer accuracy and confirmed a single band for each reaction (**Figure 2A**). DNMT1 and DNMT3a had much higher transcript levels than DNMT3b (**Figure 2B**). This is consistent with immunocytochemistry data showing that DNMT1 and DNMT3a staining was present in both neurons and glia, while staining for DNMT3b was restricted to the neuronal population (**Figure 1**). These experiments confirmed that DNMTs are expressed in rat DRG at the level of both mRNA and protein.

### UPREGULATION OF DNMT’s IN THE SNI MODEL OF NEUROPATHIC PAIN

**FIGURE 3:**
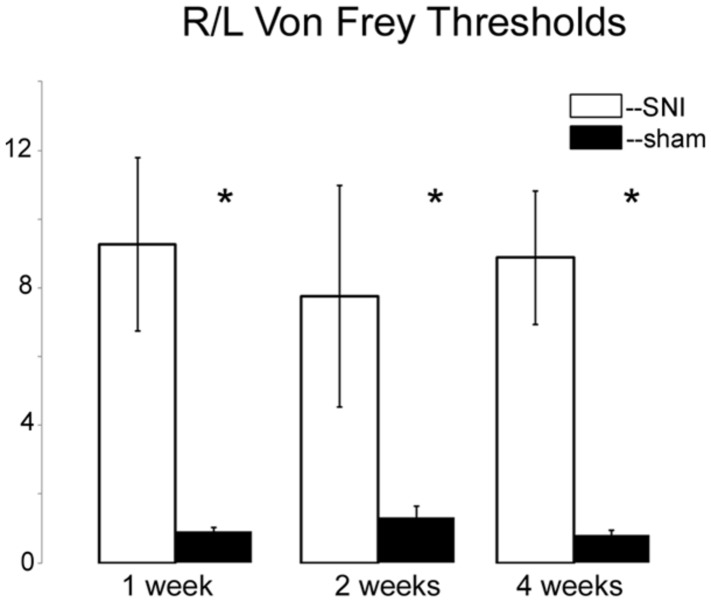
**Von Frey measurements in SNI and sham rats.** The spared nerve injury (SNI) model of neuropathic pain was used. The left or ipsilateral nerve was injured while the right or contralateral nerve was intact. Tactile sensitivity of the spared region of the operated paws was measured from the withdrawal responses to mechanical stimulation with von Frey filaments. 50% response thresholds were calculated according to Chaplan et al. (1994). Data are shown by dividing the contralateral/right paw over the ipsilateral/left paw, at 1, 2, and 4 weeks post SNI or sham surgery. All animals that underwent SNI surgery developed significant tactile allodynia in the ipsilateral/left paw that persisted until they were sacrificed, while sham rats showed no changes, *p < 0.01. *n* = 8 SNI and sham for 1 week, 4 for 2 weeks, and 6 for 4 weeks. The left foot von Frey thresholds for sham-operated and SNI rats, respectively, were 3.93 ± 0.58 g and 0.55 ± 0.41 g at 1 week, 5.16 ± 0.49 g and 0.64 ± 0.16 g at 2 weeks, and 7.93 ± 0.77 g and 0.97 ± 0.44 g at 4 weeks after surgery.

Having found that all DNMTs are expressed in the adult DRG, although in a cell-type dependent pattern, we wondered whether their expression levels are affected following neuropathic injury. Widespread changes in gene expression have been reported in animal models of neuropathic pain, and it is possible epigenetic mechanisms may underlie at least some of these changes. To test this hypothesis we took advantage of the SNI model, a robust and well-established model of neuropathic pain, which involves severing the tibial and peroneal portions of the sciatic nerve (Decosterd and Woolf, 2000). Tactile sensitivity was measured to verify the efficacy of the surgery. von-Frey filament testing was performed on all SNI and sham operated rats prior to experimental use and confirmed the presence of allodynia in all SNI rats but not sham controls. The left (operated) paw von Frey thresholds were 3.93 ± 0.58 g and 0.55 ± 0.41 g at 1 week, 5.16 ± 0.49 g and 0.64 ± 0.16 g at 2 weeks, and 7.93 ± 0.77 g and 0.97 ± 0.44 g at 4 weeks after surgery for sham-operated and SNI rats, respectively (**Figure 3**). qRT-PCR experiments were done 1, 2, and 4 weeks post surgery using L4 and L5 DRG tissue of control (sham operated) and SNI rats; genes of interest were normalized to the GAPDH reference gene. All data are shown normalized to age matched sham controls. First, the amount of beta actin transcript was quantified relative to GAPDH for all SNI and sham groups to test the stability of GAPDH and verify it’s suitability for use as a reference gene. No significant changes were detected in beta actin transcript levels between SNI and sham at 1, 2, or 4 weeks post surgery, thus confirming its suitability as a reference gene (data not shown).

**FIGURE 4:**
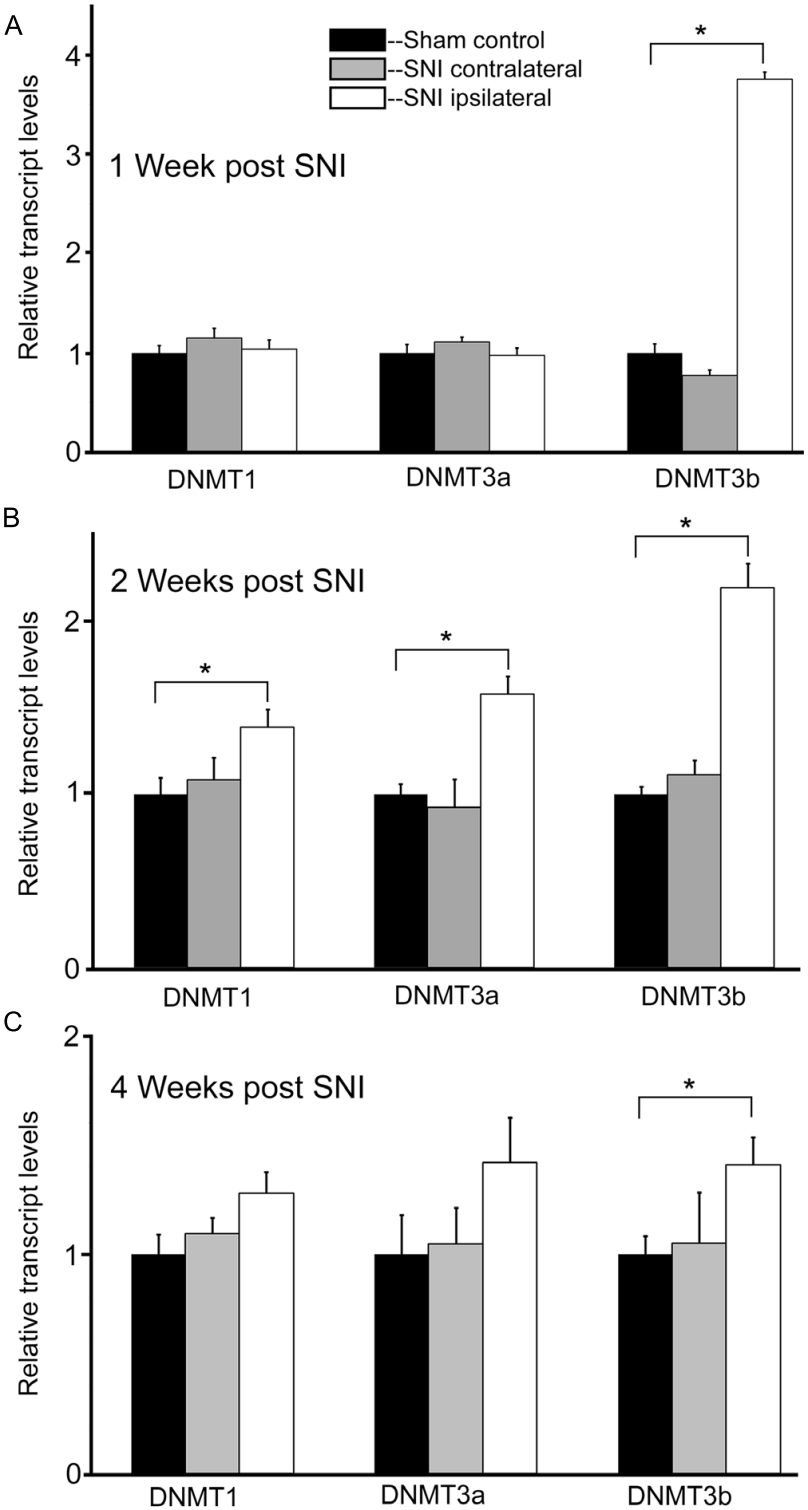
**Nerve injury induces a robust and long lasting increase in DNMT3b expression.** Gene expression is normalized to GAPDH. All SNI data are normalized to sham controls. **(A)** At 1 week post nerve injury, qRT-PCR experiments demonstrate a significant, almost fourfold increase in DNMT3b transcript in the DRG ipsilateral to the injured nerve. DRGs contralateral to nerve injury showed no significant changes, *p < 0.001. *n* = 8 SNI and 8 sham. **(B)** At 2 weeks post nerve injury, qRT-PCR experiments demonstrate a significant increase in DNMT1 (38%), DNMT3a (58%), and DNMT3b (twofold) transcripts in the DRGs ipsilateral to the injured nerve. DRGs contralateral to nerve injury showed no changes, *p < 0.05. *n* = 4 SNI and 4 sham animals. **(C)** At 4 weeks post nerve injury, qRT-PCR experiments demonstrate a significant, approximately 40% increase in DNMT3b transcripts in the DRGs ipsilateral to the injured nerve. DRGs contralateral to nerve injury showed no changes. For DRGs ipsilateral to injury in SNI rats, DNMT1 showed a trend toward being increased by 27% with *p* = 0.09 while DNMT3a also showed a slight trend of being increased by 42% with *p* = 0.22, *p < 0.05. *n* = 6 SNI and 6 sham.

At 1 week post injury, qRT-PCR experiments for DNMT transcripts demonstrated no significant changes in expression levels for both DNMT1 and DNMT3a transcripts, in the DRGs ipsi- or contralateral to the injured nerve relative to sham controls (**Figure 4A**). However, analysis of the DNMT3b transcript in the DRG ipsilateral to the injured nerve revealed a significant, almost fourfold increase (*p* < 0.001) in expression levels relative to sham (**Figure 4A**). DNMT3b expression was unchanged in the DRG contralateral to nerve injury.

In order to better understand the changes occurring in DRG neurons during more chronic conditions, DNMT transcript levels were also measured at 2 and 4 weeks post surgery. Interestingly, at 2 weeks post injury DNMT1 transcript was increased by 38% (*p* < 0.05) relative to sham controls in the DRG ipsilateral to nerve injury (**Figure 4B**). Also, DNMT3a transcript was increased by 58% (*p* < 0.05) relative to sham in the DRG ipsilateral to nerve injury. For both DNMT1 and DNMT3a, no significant changes were found in the DRG contralateral to nerve lesion. In addition DNMT3b expression was still increased more than twofold (*p* < 0.05) in the DRG ipsilateral to nerve injury, with no change contralateral to the injury (**Figure 4B**).

**FIGURE 5:**
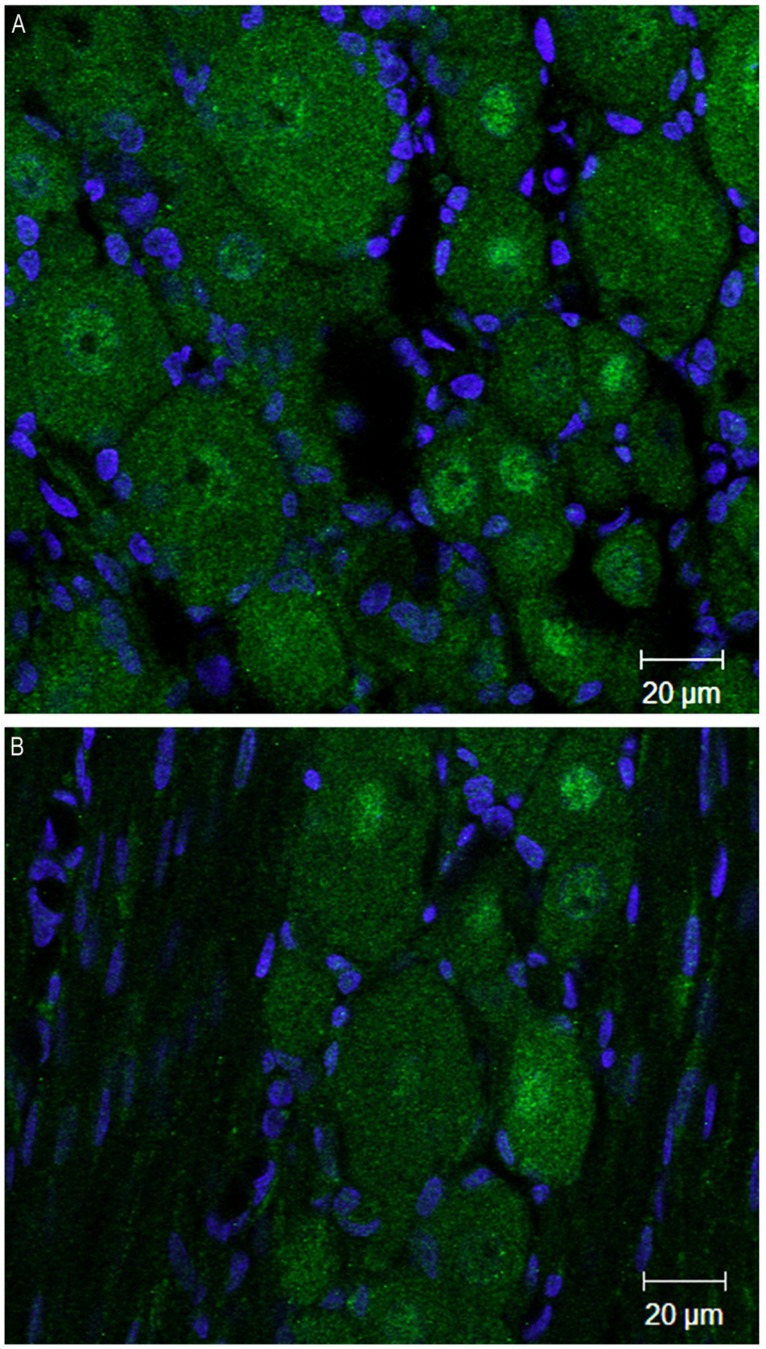
**DNMT3b transcript expression remains exclusively neuronal after peripheral nerve injury.** Immunohistochemisry was performed on sections obtained from DRG ipsilateral to the peripheral injury in two SNI rats 1 week post-surgery. **(A,B)** are two examples of images obtained by staining the DRG sections with DNMT3b (green) antibody and DAPI (blue). Note that, similar to the naïve tissue, DNMT3b expression is ubiquitous in neurons, but absent from glia.

At 4 weeks post injury, in the ipsilateral DRG both DNMT1 and DNMT3a transcripts still showed a trend for a slight increase relative to sham controls (28% for DNMT1, *p* = 0.09, 42% for DNMT3a, *p* = 0.22); no significant increase was found in the DRG contralateral to nerve injury (**Figure 4C**). DNMT3b transcript was still significantly elevated by 41% in the DRG ipsilateral to nerve injury (*p* < 0.05). DRGs contralateral to injury showed no changes. Thus, these data demonstrate that nerve injury associated changes in DNMT3b expression are early and robust, while changes in DNMT1 and DNMT3a occur later and more transiently. Thus, only DNMT3b transcript expression was significantly increased at all time points after surgery. Because, in control conditions, this transcript is found in all neurons, but not in glia, we wanted to verify whether this qualitative pattern is maintained after peripheral nerve injury. Therefore, immunohistochemistry was performed on DRG sections from 2 SNI animals 1 week after the surgery, when the transcript expression was the highest (**Figure 5**). We found that, similar to the naïve rat condition, DNMT3b expression was still neuronal-specific and no evidence for glial expression was found.

## DISCUSSION

### DNA METHYLATION IN ADULT ANIMALS

While the role of DNMTs during development has been the focus of intense study for many years (Lyko et al., 1999; Okano et al., 1999; Ko et al., 2005), much less is known about their role in the adult. However, an increasing amount of data supports a critical role for DNA methylation in adult animals, and in the brain in particular. For instance, changes in DNA methylation have been linked to long-lasting alteration of local circuits, such as those implicated in memory consolidation (Miller and Sweatt, 2007; Lubin et al., 2008). Furthermore, conditional knockdown of DNMT1 and DNMT3a in adult mouse forebrain excitatory neurons resulted in disruption of long term plasticity in area CA1, including deficits in learning and memory, and changes in gene expression and DNA methylation (Feng et al., 2010). This confirmed the crucial role played by DNMTs in adult post-mitotic neurons. Changes in DNMT gene expression as well as DNA methylation have also been shown to take place in psychiatric diseases such as schizophrenia (Tremolizzo et al., 2002; Veldic et al., 2005). Observations have also been made that involve other epigenetic mechanisms, such as histone acetylation that has been shown to be implicated in numerous brain activities such as memory formation (Fischer et al., 2007; LaPlant et al., 2010); and neurodegenerative diseases such as Huntington’s disease (Butler and Bates, 2006). Thus, strong evidence supports a critical role for epigenetic regulation of CNS activity in adult animals. So far, however, no reports have identified whether DNMT transcripts are expressed in adult DRGs and if they may be affected in the setting of neuropathic pain. Here we used immunohistochemical analysis to identify expression of DNMT1, 3a and 3b in adult rat DRG and show that all three DNMTs are expressed, although at different levels and in a cell-type dependent manner. We also demonstrate significant upregulation of DNMT’s in an animal model of neuropathic pain.

### DRG DNA METHYLATION AND PAIN

A major component of pain suffering in modern societies is chronic in nature; yet, there is little understanding regarding the mechanisms of pain chronification and no scientifically validated therapies for such conditions (Dworkin, 2002). DRGs are the first step in the pain pathway. Small and medium size DRG neurons generate c- and Aβ and Aδ nociceptor fibers. Animal as well as human studies have recently shown that all major stations in the pain pathway are affected in chronic pain conditions. In particular, pain-associated functional and/or morphological changes have been described in DRG neurons (Devor and Wall, 1990; Kajander et al., 1992; Jimenez-Andrade et al., 2006), spinal cord (Coull et al., 2003; Braz et al., 2012) and several brain areas including the amygdala (Neugebauer and Li, 2003; Neugebauer et al., 2003; Bird et al., 2005), the anterior cingulate cortex (Li et al., 2010), the hippocampus (Ren et al., 2011; Mutso et al., 2012) and the prefrontal cortex (Metz et al., 2009; Baliki et al., 2010, 2012). Critical questions that remain unanswered concern the site of origin for these changes and their temporal evolution. Epigenetic mechanisms have been previously suggested to contribute to the development of persistent pain. For instance, epigenetic suppression of GAD65 expression in the nucleus raphe magnus has been shown to favor the development of persistent inflammatory pain (following injection of complete Freund’s adjuvant; Zhang et al., 2011b). In addition levels of DNMT’s were altered in the spinal cord after nerve or inflammatory injury (Tochiki et al., 2012). Also, intrathecal spinal injection of the DNMT inhibitor 5-azacytidine was shown to alleviate neuropathic pain in the CCI model (Wang et al., 2011). Thus, we investigated the expression level of DNMT transcripts in DRGs in the SNI model of neuropathic pain. We found that the expression level of DNMT3b transcript, which in adult DRGs is expressed in neurons but not in glia, is dramatically increased at 1, 2, and 4 weeks after the neuropathic lesion. In addition, DNMT1 and DNMT3a were upregulated at 2 weeks post injury. This upregulation was at least in part surprising because neuropathic pain is associated with increased neuronal firing and neuronal depolarization was previously reported to reduce DNMT expression (Sharma et al., 2008). Therefore, the observed changes in DNMT expression may be considered the result of a complex program rather than a direct consequence of neuronal electrophysiological activity. Immunohistochemistry of SNI DRGs for DNMT3b revealed no detection of DNMT3b in glia, suggesting that glial activation following SNI surgery is not linked to DNMT3b expression, suggesting that the increase occurs in neurons. Independent of the specific mechanisms, however, altered DNMT expression in DRGs may be involved in the transition from acute to chronic pain and may underlie some of the numerous changes in gene expression in pain models. Accordingly, microarray studies carried out on DRGs from peripheral axotomy pain models have reported gene expression changes for several hundred genes (Costigan et al., 2002; Xiao et al., 2002).

Epigenetic control of genes includes both histone modifications and DNA methylation and regulates gene expression by changing the accessibility of transcriptional machinery to the DNA. Although there are some exceptions, DNA methylation of gene promoters is generally associated with gene silencing (Wolffe and Matzke, 1999). The upregulation of DNMT’s in the DRG may result in a number of specific and/or broader functional consequences. Considered broadly, one could expect upregulation of DNMTs to largely result in suppression of gene expression. Also, as DNMT3a and DNMT3b are involved primarily in *de novo* methylation (Okano et al., 1999), their upregulation may lead to new patterns of DNA methylation in DRGs. We show here robust upregulation of DNMT3b up to 4 weeks post injury, however, as DNA methylation marks can be quite stable, even the transient upregulation of DNMT1 and DNMT3a (at 2 weeks post injury) may contribute to long lasting changes in gene expression. It is worth noting that DNMT’s can also repress transcription independent of DNA methylation by recruiting the chromatin remodeling enzymes histone deacetylases (HDAC’s). DNMT1 can recruit HDAC 1 and HDAC 2 (Rountree et al., 2000) while the DNMT3 family can interact with HDAC1 via a different mechanism (Bachman et al., 2001). The upregulation of DNMTs reported here is thus consistent with recent reports of reduced histone acetylation at the promoter of several genes in a mouse model of neuropathic pain (Uchida et al., 2010a,b) and points to the possibility that inhibiting DNMT activity in the DRG after nerve injury may serve to prevent some of the pathological changes in gene expression and improve the pain phenotype.

The observation that DNMT3b is most heavily regulated in the pain condition is intriguing considering that, in keeping with our finding in DRGs, DNMT3b is expressed at low levels in adult tissue, except in thyroid, testis, and bone marrow (Xie et al., 1999). Similar to our finding in the pain condition DNMT3a and DNMT3b were shown to be upregulated in area CA1 of the hippocampus after fear conditioning in rats, and DNMT inhibitors were shown to block learning and memory. These observations support a function for DNMT3s in plastic mechanisms in adult brain tissue (Miller and Sweatt, 2007). DRG in particular may undergo plastic changes both in pathological conditions where nerve injury induces maladaptive responses in gene expression and neuronal excitability, and in physiological conditions where nociceptors receive and integrate numerous inputs from the periphery. Interestingly, DNMTs undergo alternative splicing in a tissue and developmental stage specific pattern, and DNMT3b has by far the most with some estimates of almost 40 splice variants (Okano et al., 1998; Wang et al., 2006a,b; Ostler et al., 2007; Gopalakrishnan et al., 2009; Gordon et al., 2013). It is possible that one of these developmental variants may be upregulated in the DRG following the SNI injury although future studies will be required to examine this more closely.

Finally, it is important to stress that neither the effect of neuropathic injury on DRG DNMT expression nor the pattern of DNMT expression in adult DRGs were previously investigated. Thus, as almost every study, and work addressing new ideas in particular, this paper has also limitations. One such limitation is that the functional consequences of the dysregulation in DNMT expression are unclear. Methylation array data from SNI and sham DRGs may help answer this question, but there are technical problems for such a study. The major problem consists in the difficulty to separate glia and neurons. Our data show that expression of DNMT3b, which is the lowest in DRGs and is selective for neurons, is mostly altered following neuropathic injury. The DRG contains far more glial cells than neurons, thus methylation arrays from DRG tissue would in reality provide more of a glial methylation signature than neuronal. Considering that high cost of a global DNA methylation analysis and this lack of cell specificity the cost to benefit ratio does not justify such an experiment at this moment although one may imagine that in the near future cell-sorting capability may render such analysis worthwhile.

## Conflict of Interest Statement

The authors declare that the research was conducted in the absence of any commercial or financial relationships that could be construed as a potential conflict of interest.

## References

[B1] Bachman K. E. Rountree M. R. Baylin S. B. (2001). Dnmt3a and Dnmt3b are transcriptional repressors that exhibit unique localization properties to heterochromatin. *J. Biol. Chem.* 276 32282–32287 10.1074/jbc.M104661200 11427539

[B2] Baliki M. N. Geha P. Y. Fields H. L. Apkarian A. V. (2010). Predicting value of pain and analgesia: nucleus accumbens response to noxious stimuli changes in the presence of chronic pain. *Neuron* 66 149–160 10.1016/j.neuron.2010.03.002 20399736 PMC2873199

[B3] Baliki M. N. Petre B. Torbey S. Herrmann K. M. Huang L. Schnitzer T. J. (2012). Corticostriatal functional connectivity predicts transition to chronic back pain. *Nat. Neurosci.* 15 1117–1119 10.1038/nn.3153 22751038 PMC3411898

[B4] Barreto G. Schafer A. Marhold J. Stach D. Swaminathan S. K. Handa V. (2007). Gadd45a promotes epigenetic gene activation by repair-mediated DNA demethylation. *Nature* 445 671–675 10.1038/nature05515 17268471

[B5] Bestor T. Laudano A. Mattaliano R. Ingram V. (1988). Cloning and sequencing of a cDNA encoding DNA methyltransferase of mouse cells. The carboxyl-terminal domain of the mammalian enzymes is related to bacterial restriction methyltransferases. *J. Mol. Biol.* 203 971–983 10.1016/0022-2836(88)90122-2 3210246

[B6] Bird G. C. Lash L. L. Han J. S. Zou X. Willis W. D. Neugebauer V. (2005). Protein kinase A-dependent enhanced NMDA receptor function in pain-related synaptic plasticity in rat amygdala neurones. *J. Physiol.* 564 907–921 10.1113/jphysiol.2005.084780 15760935 PMC1464474

[B7] Braz J. M. Sharif-Naeini R. Vogt D. Kriegstein A. Alvarez-Buylla A. Rubenstein J. L. (2012). Forebrain GABAergic neuron precursors integrate into adult spinal cord and reduce injury-induced neuropathic pain. *Neuron* 74 663–675 10.1016/j.neuron.2012.02.033 22632725 PMC3361692

[B8] Bruniquel D. Schwartz R. H. (2003). Selective, stable demethylation of the interleukin-2 gene enhances transcription by an active process. *Nat. Immunol.* 4 235–240 10.1038/ni887 12548284

[B9] Bustin S. A. Beaulieu J. F. Huggett J. Jaggi R. Kibenge F. S. Olsvik P. A. (2010). MIQE precis: practical implementation of minimum standard guidelines for fluorescence-based quantitative real-time PCR experiments. *BMC Mol. Biol.* 11:74 10.1186/1471-2199-11-74 PMC2955025 20858237

[B10] Butler R. Bates G. P. (2006). Histone deacetylase inhibitors as therapeutics for polyglutamine disorders. *Nat. Rev. Neurosci.* 7 784–796 10.1038/nrn1989 16988654

[B11] Chaplan S. R. Bach F. W. Pogrel J. W. Chung J. M. Yaksh T. L. (1994). Quantitative assessment of tactile allodynia in the rat paw. *J. Neurosci. Methods* 53 55–63 10.1016/0165-0270(94)90144-9 7990513

[B12] Chedin F. (2011). The DNMT3 family of mammalian de novo DNA methyltransferases. *Prog. Mol. Biol. Transl. Sci.* 101 255–285 10.1016/B978-0-12-387685-0.00007-X 21507354

[B13] Costigan M. Befort K. Karchewski L. Griffin R. S. D’urso D. Allchorne A. (2002). Replicate high-density rat genome oligonucleotide microarrays reveal hundreds of regulated genes in the dorsal root ganglion after peripheral nerve injury. *BMC Neurosci.* 3:16 10.1186/1471-2202-3-16 PMC139981 12401135

[B14] Coull J. A. Boudreau D. Bachand K. Prescott S. A. Nault F. Sik A. (2003). Trans-synaptic shift in anion gradient in spinal lamina I neurons as a mechanism of neuropathic pain. *Nature* 424 938–942 10.1038/nature01868 12931188

[B15] Decosterd I. Woolf C. J. (2000). Spared nerve injury: an animal model of persistent peripheral neuropathic pain. *Pain* 87 149–158 10.1016/S0304-3959(00)00276-1 10924808

[B16] Devor M. Wall P. D. (1990). Cross-excitation in dorsal root ganglia of nerve-injured and intact rats. *J. Neurophysiol.* 64 1733–1746 2074461 10.1152/jn.1990.64.6.1733

[B17] Dib-Hajj S. D. Fjell J. Cummins T. R. Zheng Z. Fried K. Lamotte R. (1999). Plasticity of sodium channel expression in DRG neurons in the chronic constriction injury model of neuropathic pain. *Pain* 83 591–600 10.1016/S0304-3959(99)00169-4 10568868

[B18] Di Giaimo R. Russo G. M. Bevilacqua M. A. Iovine B. Del Gaudio R. Geraci G. (2005). The expression of de novo DNA methylase DNMT3b, of the methyl-CpG binding protein MBD2b and of 5-MCDG glycosylase shows two waves of induction during CaCO-2 cell differentiation. *Gene* 351 73–81 10.1016/j.gene.2005.02.015 15823509

[B19] Dupont C. Gribnau J. (2013). Different flavors of X-chromosome inactivation in mammals. *Curr. Opin. Cell Biol.* 25 314–321 10.1016/j.ceb.2013.03.001 23578369

[B20] Dworkin R. H. (2002). An overview of neuropathic pain: syndromes, symptoms, signs, and several mechanisms. *Clin. J. Pain* 18 343–349 10.1097/00002508-200211000-00001 12441827

[B21] Ehrlich M. (2003). Expression of various genes is controlled by DNA methylation during mammalian development. *J. Cell. Biochem.* 88 899–910 10.1002/jcb.10464 12616529

[B22] Fan N. Sikand P. Donnelly D. F. Ma C. Lamotte R. H. (2011). Increased Na^+^ and K^+^ currents in small mouse dorsal root ganglion neurons after ganglion compression. *J. Neurophysiol.* 106 211–218 10.1152/jn.00065.2011 21525373 PMC3295375

[B23] Feng J. Zhou Y. Campbell S. L. Le T. Li E. Sweatt J. D. (2010). Dnmt1 and Dnmt3a maintain DNA methylation and regulate synaptic function in adult forebrain neurons. *Nat. Neurosci.* 13 423–430 10.1038/nn.2514 20228804 PMC3060772

[B24] Fischer A. Sananbenesi F. Wang X. Dobbin M. Tsai L. H. (2007). Recovery of learning and memory is associated with chromatin remodelling. *Nature* 447 178–182 10.1038/nature05772 17468743

[B25] Gardner E. P. Martin J. H. Jessell T. M. (2000). “The bodily senses,” in *Principles of Neural Science* eds Jessell T. M. Kandel E. R. Schwartz J. H. (New York: McGraw-Hill).

[B26] Gendrel A. V. Apedaile A. Coker H. Termanis A. Zvetkova I. Godwin J. (2012). Smchd1-dependent and -independent pathways determine developmental dynamics of CpG island methylation on the inactive X chromosome. *Dev. Cell* 23 265–279 10.1016/j.devcel.2012.06.011 22841499 PMC3437444

[B27] Gopalakrishnan S. Van Emburgh B. O. Shan J. Su Z. Fields C. R. Vieweg J. (2009). A novel DNMT3B splice variant expressed in tumor and pluripotent cells modulates genomic DNA methylation patterns and displays altered DNA binding. *Mol. Cancer Res.* 7 1622–1634 10.1158/1541-7786.MCR-09-0018 19825994 PMC2783805

[B28] Gordon C. A. Hartono S. R. Chedin F. (2013). Inactive DNMT3B splice variants modulate de novo DNA methylation. *PLoS ONE* 8:e69486 10.1371/journal.pone.0069486 PMC3716610 23894490

[B29] Grayson D. R. Jia X. Chen Y. Sharma R. P. Mitchell C. P. Guidotti A. (2005). Reelin promoter hypermethylation in schizophrenia. *Proc. Natl. Acad. Sci. U.S.A.* 102 9341–9346 10.1073/pnas.0503736102 15961543 PMC1166626

[B30] Jimenez-Andrade J. M. Peters C. M. Mejia N. A. Ghilardi J. R. Kuskowski M. A. Mantyh P. W. (2006). Sensory neurons and their supporting cells located in the trigeminal, thoracic and lumbar ganglia differentially express markers of injury following intravenous administration of paclitaxel in the rat. *Neurosci. Lett.* 405 62–67 10.1016/j.neulet.2006.06.043 16854522

[B31] Kaas G. A. Zhong C. Eason D. E. Ross D. L. Vachhani R. V. Ming G. L. (2013). TET1 controls CNS 5-methylcytosine hydroxylation, active DNA demethylation, gene transcription, and memory formation. *Neuron* 79 1086–1093 10.1016/j.neuron.2013.08.032 24050399 PMC3816951

[B32] Kajander K. C. Wakisaka S. Bennett G. J. (1992). Spontaneous discharge originates in the dorsal root ganglion at the onset of a painful peripheral neuropathy in the rat. *Neurosci. Lett.* 138 225–228 10.1016/0304-3940(92)90920-3 1319012

[B33] Ko Y. G. Nishino K. Hattori N. Arai Y. Tanaka S. Shiota K. (2005). Stage-by-stage change in DNA methylation status of Dnmt1 locus during mouse early development. *J. Biol. Chem.* 280 9627–9634 10.1074/jbc.M413822200 15634679

[B34] LaPlant Q. Vialou V. Covington H. E. III Dumitriu D. Feng J. Warren B. L. (2010). Dnmt3a regulates emotional behavior and spine plasticity in the nucleus accumbens. *Nat. Neurosci.* 13 1137–1143 10.1038/nn.2619 20729844 PMC2928863

[B35] Li X. Y. Ko H. G. Chen T. Descalzi G. Koga K. Wang H. (2010). Alleviating neuropathic pain hypersensitivity by inhibiting PKMzeta in the anterior cingulate cortex. *Science* 330 1400–1404 10.1126/science.1191792 21127255

[B36] Lubin F. D. Roth T. L. Sweatt J. D. (2008). Epigenetic regulation of BDNF gene transcription in the consolidation of fear memory. *J. Neurosci.* 28 10576–10586 10.1523/JNEUROSCI.1786-08.2008 18923034 PMC3312036

[B37] Lyko F. Ramsahoye B. H. Kashevsky H. Tudor M. Mastrangelo M. A. Orr-Weaver T. L. (1999). Mammalian (cytosine-5) methyltransferases cause genomic DNA methylation and lethality in *Drosophila*. *Nat. Genet.* 23 363–366 10.1038/15551 10545955

[B38] McGowan P. O. Sasaki A. D’alessio A. C. Dymov S. Labonte B. Szyf M. (2009). Epigenetic regulation of the glucocorticoid receptor in human brain associates with childhood abuse. *Nat. Neurosci.* 12 342–348 10.1038/nn.2270 19234457 PMC2944040

[B39] Metivier R. Gallais R. Tiffoche C. Le Peron C. Jurkowska R. Z. Carmouche R. P. (2008). Cyclical DNA methylation of a transcriptionally active promoter. *Nature* 452 45–50 10.1038/nature06544 18322525

[B40] Metz A. E. Yau H. J. Centeno M. V. Apkarian A. V. Martina M. (2009). Morphological and functional reorganization of rat medial prefrontal cortex in neuropathic pain. *Proc. Natl. Acad. Sci. U.S.A.* 106 2423–2428 10.1073/pnas.0809897106 19171885 PMC2650172

[B41] Michaelis M. Devor M. Janig W. (1996). Sympathetic modulation of activity in rat dorsal root ganglion neurons changes over time following peripheral nerve injury. *J. Neurophysiol.* 76 753–763 8871196 10.1152/jn.1996.76.2.753

[B42] Miller C. A. Gavin C. F. White J. A. Parrish R. R. Honasoge A. Yancey C. R. (2010). Cortical DNA methylation maintains remote memory. *Nat. Neurosci.* 13 664–666 10.1038/nn.2560 20495557 PMC3043549

[B43] Miller C. A. Sweatt J. D. (2007). Covalent modification of DNA regulates memory formation. *Neuron* 53 857–869 10.1016/j.neuron.2007.02.022 17359920

[B44] Mutso A. A. Radzicki D. Baliki M. N. Huang L. Banisadr G. Centeno M. V. (2012). Abnormalities in hippocampal functioning with persistent pain. *J. Neurosci.* 32 5747–5756 10.1523/JNEUROSCI.0587-12.2012 22539837 PMC3365570

[B45] Neugebauer V. Li W. (2003). Differential sensitization of amygdala neurons to afferent inputs in a model of arthritic pain. *J. Neurophysiol.* 89 716–727 10.1152/jn.00799.2002 12574449

[B46] Neugebauer V. Li W. Bird G. C. Bhave G. Gereau R. W. T. (2003). Synaptic plasticity in the amygdala in a model of arthritic pain: differential roles of metabotropic glutamate receptors 1 and 5. *J. Neurosci.* 23 52–63 12514201 10.1523/JNEUROSCI.23-01-00052.2003 PMC6742141

[B47] Nordin M. Nystrom B. Wallin U. Hagbarth K. E. (1984). Ectopic sensory discharges and paresthesiae in patients with disorders of peripheral nerves, dorsal roots and dorsal columns. *Pain* 20 231–245 10.1016/0304-3959(84)90013-7 6096790

[B48] Novakovic S. D. Tzoumaka E. Mcgivern J. G. Haraguchi M. Sangameswaran L. Gogas K. R. (1998). Distribution of the tetrodotoxin-resistant sodium channel PN3 in rat sensory neurons in normal and neuropathic conditions. *J. Neurosci.* 18 2174–2187 9482802 10.1523/JNEUROSCI.18-06-02174.1998 PMC6792911

[B49] Okano M. Bell D. W. Haber D. A. Li E. (1999). DNA methyltransferases Dnmt3a and Dnmt3b are essential for de novo methylation and mammalian development. *Cell* 99 247–257 10.1016/S0092-8674(00)81656-6 10555141

[B50] Okano M. Xie S. Li E. (1998). Cloning and characterization of a family of novel mammalian DNA (cytosine-5) methyltransferases. *Nat. Genet.* 19 219–220 10.1038/890 9662389

[B51] Ooi S. K. Bestor T. H. (2008). The colorful history of active DNA demethylation. *Cell* 133 1145–1148 10.1016/j.cell.2008.06.009 18585349

[B52] Ooi S. K. Wolf D. Hartung O. Agarwal S. Daley G. Q. Goff S. P. (2010). Dynamic instability of genomic methylation patterns in pluripotent stem cells. *Epigenetics Chromatin* 3 17 10.1186/1756-8935-3-17 PMC2954997 20868487

[B53] Ostler K. R. Davis E. M. Payne S. L. Gosalia B. B. Exposito-Cespedes J. Le Beau M. M. (2007). Cancer cells express aberrant DNMT3B transcripts encoding truncated proteins. *Oncogene* 26 5553–5563 10.1038/sj.onc.1210351 17353906 PMC2435620

[B54] Qin W. Leonhardt H. Pichler G. (2011). Regulation of DNA methyltransferase 1 by interactions and modifications. *Nucleus* 2 392–402 10.4161/nucl.2.5.17928 21989236

[B55] Ren W. J. Liu Y. Zhou L. J. Li W. Zhong Y. Pang R. P. (2011). Peripheral nerve injury leads to working memory deficits and dysfunction of the hippocampus by upregulation of TNF-α in rodents. *Neuropsychopharmacology* 36 979–992 10.1038/npp.2010.236 21289602 PMC3077267

[B56] Ringkamp M. Meyer R. A. (2009). “Physiology of nociceptors,” in *Science of Pain* eds Bushnell M. C. Bausbaum A. I. (San Diego: Academic Press) 97–114

[B57] Rountree M. R. Bachman K. E. Baylin S. B. (2000). DNMT1 binds HDAC2 and a new co-repressor, DMAP1, to form a complex at replication foci. *Nat. Genet.* 25 269–277 10.1038/77023 10888872

[B58] Schmittgen T. D. Livak K. J. (2008). Analyzing real-time PCR data by the comparative C(T) method. *Nat. Protoc.* 3 1101–1108 10.1038/nprot.2008.73 18546601

[B59] Sharif J. Muto M. Takebayashi S. Suetake I. Iwamatsu A. Endo T. A. (2007). The SRA protein Np95 mediates epigenetic inheritance by recruiting Dnmt1 to methylated DNA. *Nature* 450 908–912 10.1038/nature06397 17994007

[B60] Sharma R. P. Tun N. Grayson D. R. (2008). Depolarization induces downregulation of DNMT1 and DNMT3a in primary cortical cultures. *Epigenetics* 3 74–80 10.4161/epi.3.2.6103 18536530

[B61] Sukhotinsky I. Ben-Dor E. Raber P. Devor M. (2004). Key role of the dorsal root ganglion in neuropathic tactile hypersensibility. *Eur. J. Pain* 8 135–143 10.1016/S1090-3801(03)00086-7 14987623

[B62] Szyf M. Bick J. (2013). DNA methylation: a mechanism for embedding early life experiences in the genome. *Child Dev.* 84 49–57 10.1111/j.1467-8624.2012.01793.x 22880724 PMC4039199

[B63] Tan Z. Y. Donnelly D. F. Lamotte R. H. (2006). Effects of a chronic compression of the dorsal root ganglion on voltage-gated Na^+^ and K^+^ currents in cutaneous afferent neurons. *J. Neurophysiol.* 95 1115–1123 10.1152/jn.00830.2005 16424456

[B64] Tanaka M. Cummins T. R. Ishikawa K. Dib-Hajj S. D. Black J. A. Waxman S. G. (1998). SNS Na^+^ channel expression increases in dorsal root ganglion neurons in the carrageenan inflammatory pain model. *Neuroreport* 9 967–972 10.1097/00001756-199804200-00003 9601651

[B65] Tochiki K. K. Cunningham J. Hunt S. P. Geranton S. M. (2012). The expression of spinal methyl-CpG-binding protein 2, DNA methyltransferases and histone deacetylases is modulated in persistent pain states. *Mol. Pain* 8 14 10.1186/1744-8069-8-14 PMC3351747 22369085

[B66] Tremolizzo L. Carboni G. Ruzicka W. B. Mitchell C. P. Sugaya I. Tueting P. (2002). An epigenetic mouse model for molecular and behavioral neuropathologies related to schizophrenia vulnerability. *Proc. Natl. Acad. Sci. U.S.A.* 99 17095–17100 10.1073/pnas.262658999 12481028 PMC139275

[B67] Uchida H. Ma L. Ueda H. (2010a). Epigenetic gene silencing underlies C-fiber dysfunctions in neuropathic pain. *J. Neurosci.* 30 4806–4814 10.1523/JNEUROSCI.5541-09.2010 20357131 PMC6632306

[B68] Uchida H. Sasaki K. Ma L. Ueda H. (2010b). Neuron-restrictive silencer factor causes epigenetic silencing of Kv4.3 gene after peripheral nerve injury. *Neuroscience* 166 1–4 10.1016/j.neuroscience.2009.12.021 20006971

[B69] Veldic M. Guidotti A. Maloku E. Davis J. M. Costa E. (2005). In psychosis, cortical interneurons overexpress DNA-methyltransferase 1. *Proc. Natl. Acad. Sci. U.S.A.* 102 2152–2157 10.1073/pnas.0409665102 15684088 PMC548582

[B70] Wang J. Walsh G. Liu D. D. Lee J. J. Mao L. (2006a). Expression of Delta DNMT3B variants and its association with promoter methylation of p16 and RASSF1A in primary non-small cell lung cancer. *Cancer Res.* 66 8361–8366 10.1158/0008-5472.CAN-06-2031 16951144

[B71] Wang L. Wang J. Sun S. Rodriguez M. Yue P. Jang S. J. (2006b). A novel DNMT3B subfamily, DeltaDNMT3B, is the predominant form of DNMT3B in non-small cell lung cancer. *Int. J. Oncol.* 29 201–207 16773201

[B72] Wang Y. Liu C. Guo Q. L. Yan J. Q. Zhu X. Y. Huang C. S. (2011). Intrathecal 5-azacytidine inhibits global DNA methylation and methyl- CpG-binding protein 2 expression and alleviates neuropathic pain in rats following chronic constriction injury. *Brain Res.* 1418 64–69 10.1016/j.brainres.2011.08.040 21925646

[B73] Weng X. Smith T. Sathish J. Djouhri L. (2012). Chronic inflammatory pain is associated with increased excitability and hyperpolarization-activated current (Ih) in C- but not Adelta-nociceptors. *Pain* 153 900–914 10.1016/j.pain.2012.01.019 22377439

[B74] Wolf S. F. Jolly D. J. Lunnen K. D. Friedmann T. Migeon B. R. (1984). Methylation of the hypoxanthine phosphoribosyltransferase locus on the human X chromosome: implications for X-chromosome inactivation. *Proc. Natl. Acad. Sci. U.S.A.* 81 2806–2810 10.1073/pnas.81.9.2806 6585829 PMC345159

[B75] Wolffe A. P. Matzke M. A. (1999). Epigenetics: regulation through repression. *Science* 286 481–486 10.1126/science.286.5439.481 10521337

[B76] Xiang Z. Xiong Y. Yan N. Li X. Mao Y. Ni X. (2008). Functional up-regulation of P2X3 receptors in the chronically compressed dorsal root ganglion. *Pain* 140 23–34 10.1016/j.pain.2008.07.006 18715715 PMC2667225

[B77] Xiao H. S. Huang Q. H. Zhang F. X. Bao L. Lu Y. J. Guo C. (2002). Identification of gene expression profile of dorsal root ganglion in the rat peripheral axotomy model of neuropathic pain. *Proc. Natl. Acad. Sci. U.S.A.* 99 8360–8365 10.1073/pnas.122231899 12060780 PMC123072

[B78] Xie S. Wang Z. Okano M. Nogami M. Li Y. He W. W. (1999). Cloning, expression and chromosome locations of the human DNMT3 gene family. *Gene* 236 87–95 10.1016/S0378-1119(99)00252-8 10433969

[B79] Zhang L. Lu D. Y. Ma W. Y. Li Y. (2011a). Age-related changes in the localization of DNA methyltransferases during meiotic maturation in mouse oocytes. *Fertil. Steril.* 95 1531–1534 e1. 10.1016/j.fertnstert.2010.06.050 20674893

[B80] Zhang Z. Cai Y. Q. Zou F. Bie B. Pan Z. Z. (2011b). Epigenetic suppression of GAD65 expression mediates persistent pain. *Nat. Med.* 17 1448–1455 10.1038/nm.2442 21983856 PMC3210928

[B81] Zhu Q. Wang L. Zhang Y. Zhao F. H. Luo J. Xiao Z. (2012). Increased expression of DNA methyltransferase 1 and 3a in human temporal lobe epilepsy. *J. Mol. Neurosci.* 46 420–426 10.1007/s12031-011-9602-7 21826395

